# Supplementary material for: Intestinal microbiota-derived short-chain fatty acids regulation of immune cell IL-22 production and gut immunity
Source: Nat Commun. 2020 Sep 8;11:4457. doi: 10.1038/s41467-020-18262-6 (PMC7478978; doi:10.1038/s41467-020-18262-6)
Supplement: Supplementary file 1 — Supplementary Information [file 41467_2020_18262_MOESM1_ESM.pdf]

Intestinal microbiota-derived short-chain fatty acids regulation of immune cell IL-22 production and gut immunity

Yang et al

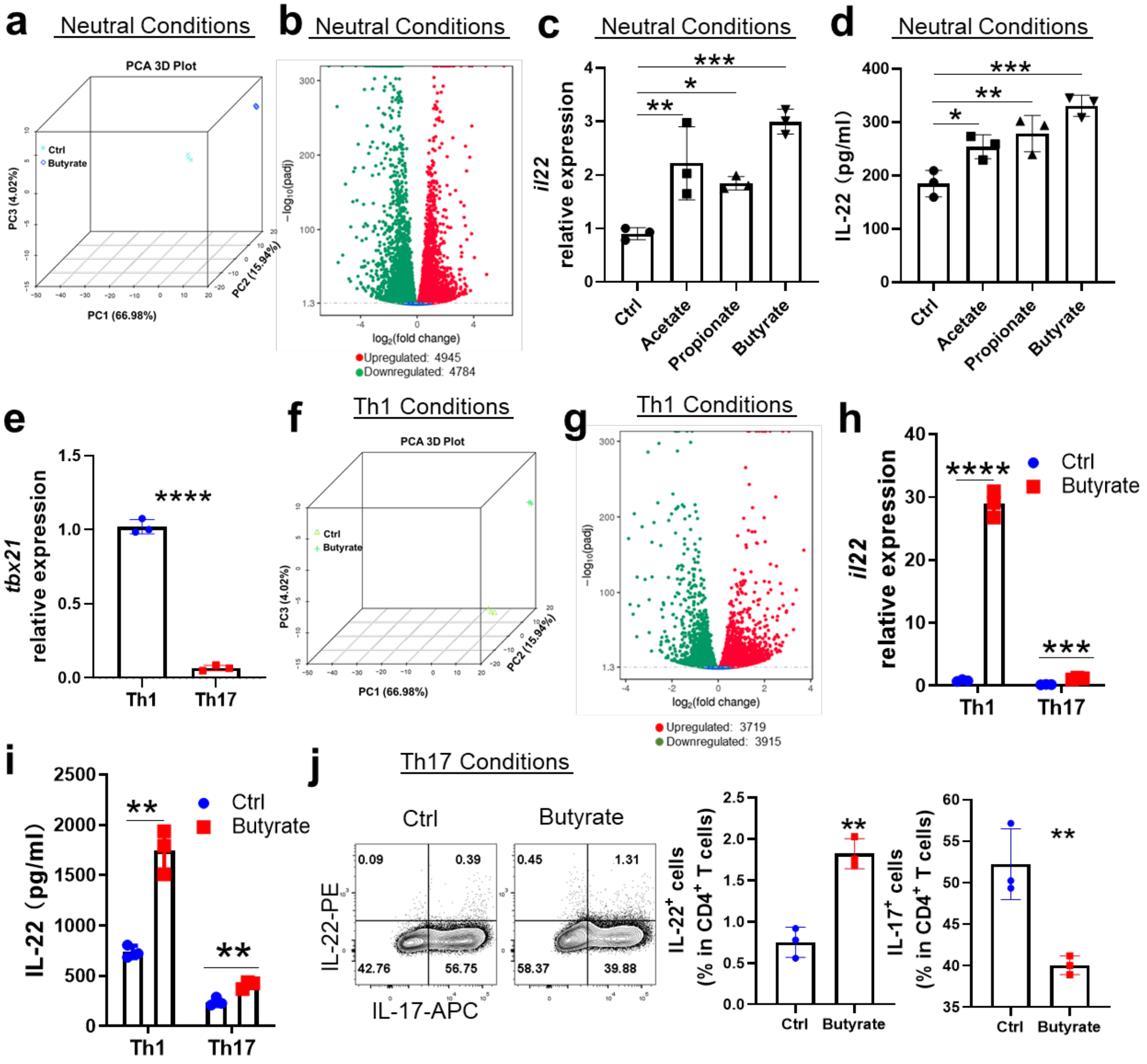

Supplementary Fig. 1. SCFAs promote CD4$^+$ T cell IL-22 production. (**a-b**) WT splenic CD4$^+$ T cells were activated with anti-CD3 and anti-CD28 mAb in the presence or absence of butyrate (0.5 mM) for 2 days (n = 3/group). RNA sequencing was performed. Principal components analysis (PCA) plot (**a**) and Volcano plot (**b**) of different gene profiles were shown. (**c-d**) WT splenic CD4$^+$ T cells were activated with anti-CD3 and anti-CD28 mAb under Th1 conditions with or without acetate (10 mM), propionate (0.5 mM), or butyrate (0.5 mM) for 2 days (n = 3/group). *il22* expression was analyzed by using qRT-PCR (**c**), and IL-22 in culture supernatants was

analyzed by using ELISA (**d**). (**e**) WT splenic CD4$^+$ T cells were activated with anti-CD3 and anti-CD28 mAb under Th1 or Th17 conditions (n= 3/group). T*bx21* expression was assessed by qRT-PCR. **(f-g)** Splenic CD4$^+$ T cells were activated with anti-CD3 and anti-CD28 mAb under Th1 conditions in the presence or absence of butyrate (0.5 mM) for 2 days (n = 3/ group). RNA sequencing was performed. PCA plot (**f**) and Volcano plot (**g**) of different gene profiles were shown. (**h-i**) CBir1 CD4$^+$ T cells were activated with APCs and 1 µg/ml CBir1 peptide under Th1 or Th17 conditions with or without butyrate (0.5 mM) for 60 h (n = 3/group). IL-22 mRNA (**h**) and protein (**i**) levels were analyzed by qRT-PCR and ELISA (**h**). (**j**) CBir1 CD4$^+$ T cells were activated under Th17 conditions with or without butyrate (0.5 mM) for 5 days (n = 3/group). IL-22 production was measured by flow cytometry. One representative of three independent experiments was shown. The data was expressed as mean ± SD. Statistical significance was tested by two-tailed one-way ANOVA (**c-d**) or two-tailed unpaired Student *t* test (**e, and h-j**). **c**, $^{**}p = 0.0063$, $^{*}p = 0.0355$, $^{***}p = 0.0003$; **d**, $^{*}p = 0.0289$, $^{**}p = 0.0057$, $^{***}p = 0.0004$; **e**, $^{****}p < 0.0001$; **h**,$^{****}p < 0.0001$, $^{***}p = 0.0004$; **i**, $^{**}p = 0.0015$ (Th1) and 0.0062 (Th17); **j**, middle panel: $^{**}p = 0.0020$; right panel: $^{**}p = 0.0088$.

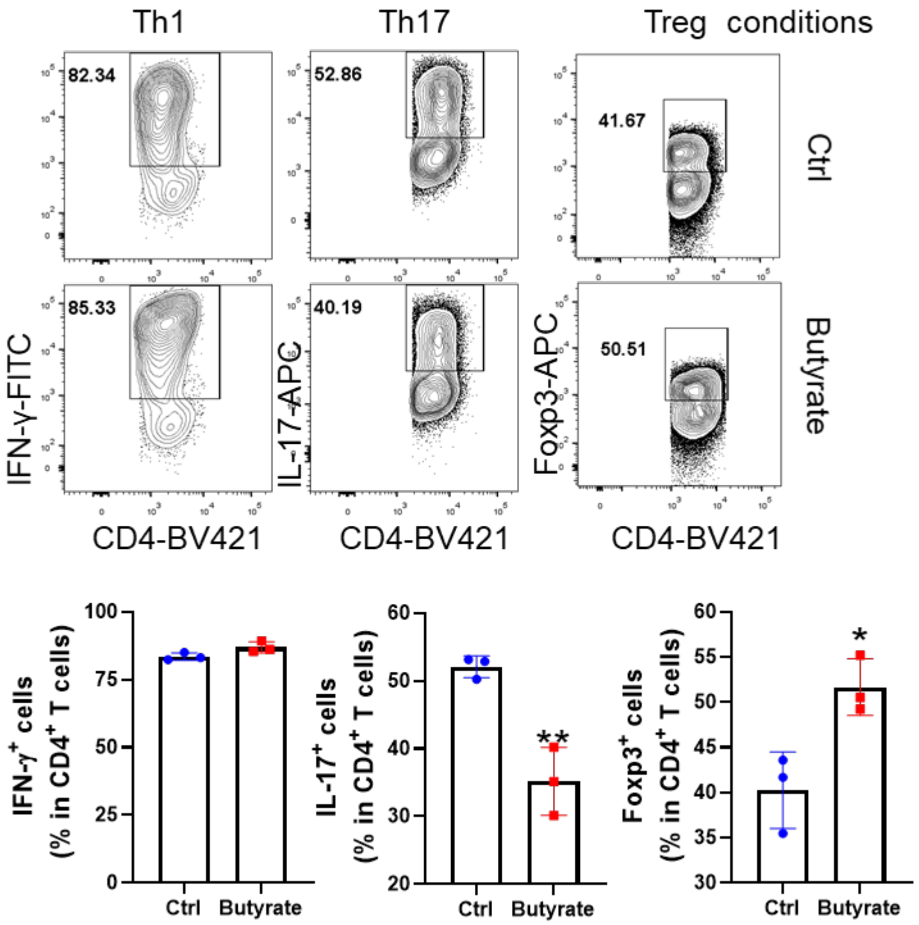

Supplementary Fig. 2. Butyrate inhibits Th17 cell differentiation but promotes Treg differentiation. CBir1 CD4$^+$ T cells were activated with APCs and CBir1 peptide under Th1, Th17, Treg conditions with butyrate (0.5 mM) (n =3/group). IFNγ, IL-17, and Foxp3 levels were measured by flow cytometry on day 5. One representative of three independent experiments was shown. The data was expressed as mean ± SD. Statistical significance was tested by two-tailed unpaired Student *t* test. ***p* = 0.0052; **p* = 0.0200.

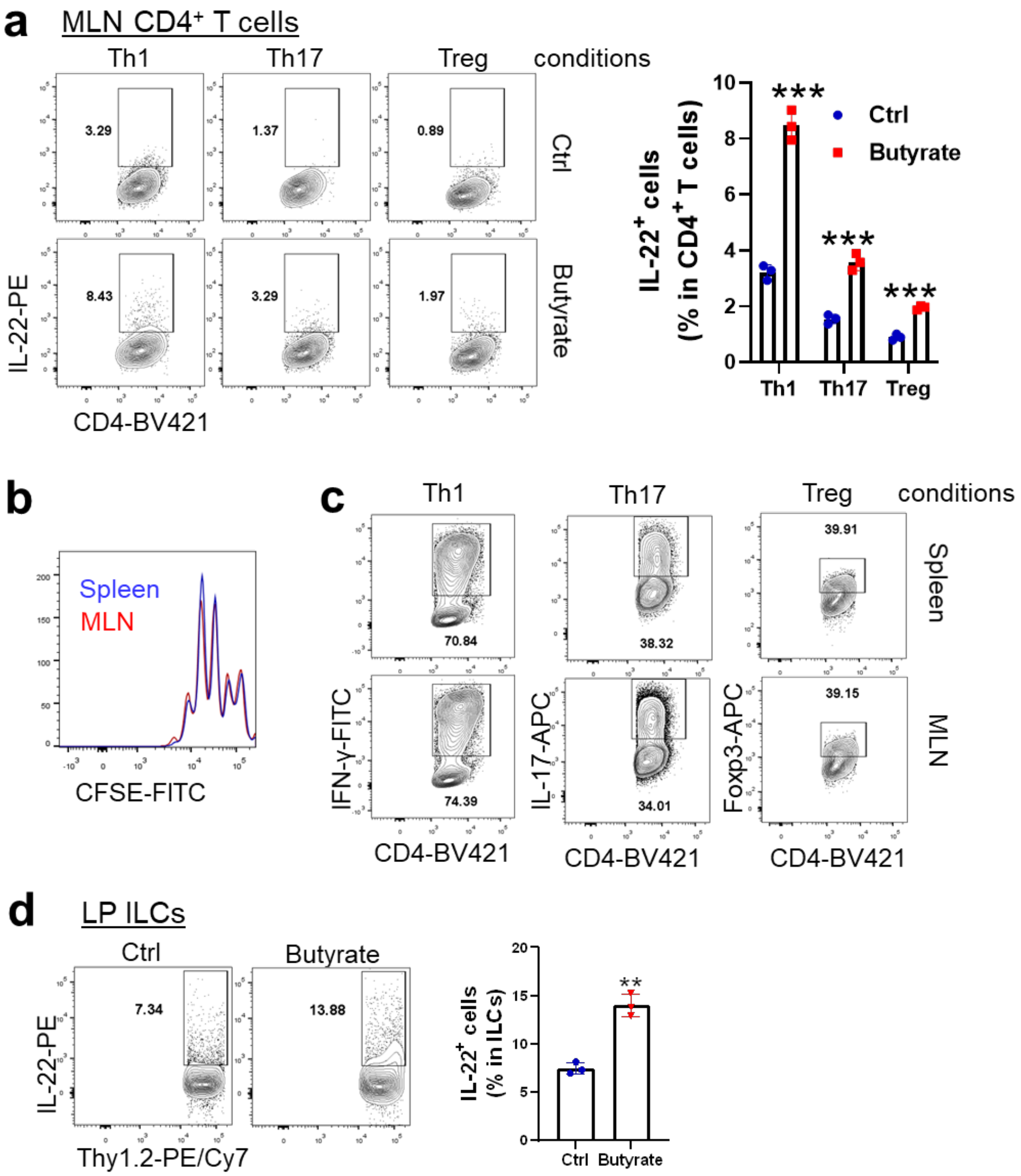

Supplementary Fig. 3. Butyrate promotes IL-22 production in MLN CD4⁺ T cells and intestinal ILCs. (**a**) CD4⁺ T cells were isolated from MLN of WT mice and activated with anti-CD3 and anti-CD28 mAb in the presence or absence of butyrate (0.5 mM) under Th1, Th17, and Treg conditions. IL-22 production was measured by flow cytometry on day 5. (**b**) WT splenic and MLN CD4⁺ T cells were labeled with CFSE and activated with anti-CD3 and anti-CD28 mAb for

3 days. CFSE dilution was measured by flow cytometry. (**c**) WT splenic and MLN CD4$^+$ T cells were activated with anti-CD3 and anti-CD28 mAb in the presence or absence of butyrate (0.5 mM) under Th1, Th17, and Treg conditions for 5 days. Cytokine levels were analyzed by flow cytometry. (**d**) Intestinal lamia propria cells were treated with IL-23 (20 ng/ml) in in the presence or absence of butyrate (0.5 mM) for 16 h (n=3/groups), and IL-22 production in ILCs were analyzed by gating on Linage$^-$ Thy1$^+$ population by flow cytometry. One representative of three independent experiments was shown. The data was expressed as mean ± SD. Statistical significance was tested by two-tailed unpaired Student *t* test. **a**, ***p = 0.0001 (Th1), 0.0005 (Th17), and 0.0002 (Treg); **d**, **p = 0.0010.

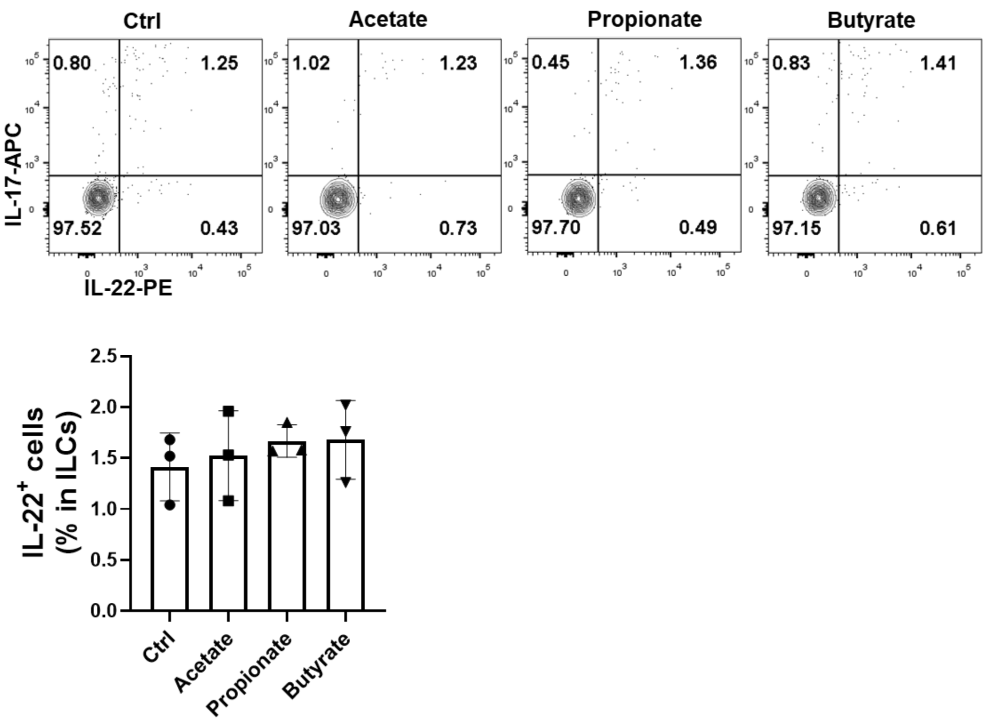

Supplementary Fig. 4. SCFAs did not affect IL-22 production in ILCs without IL-23 stimulation. CD4^{+} T cell-depleted splenic cells were treated with or without acetate (10 mM), propionate (0.5 mM), or butyrate (0.5 mM) for 16 h (n = 3/groups), and IL-22 and IL-17 production in ILCs were analyzed by gating on Linage^{-} Thy1^{+} population by flow cytometry. One representative of three independent experiments was shown. The data was expressed as mean ± SD. Statistical significance was tested by two-tailed one-way ANOVA.

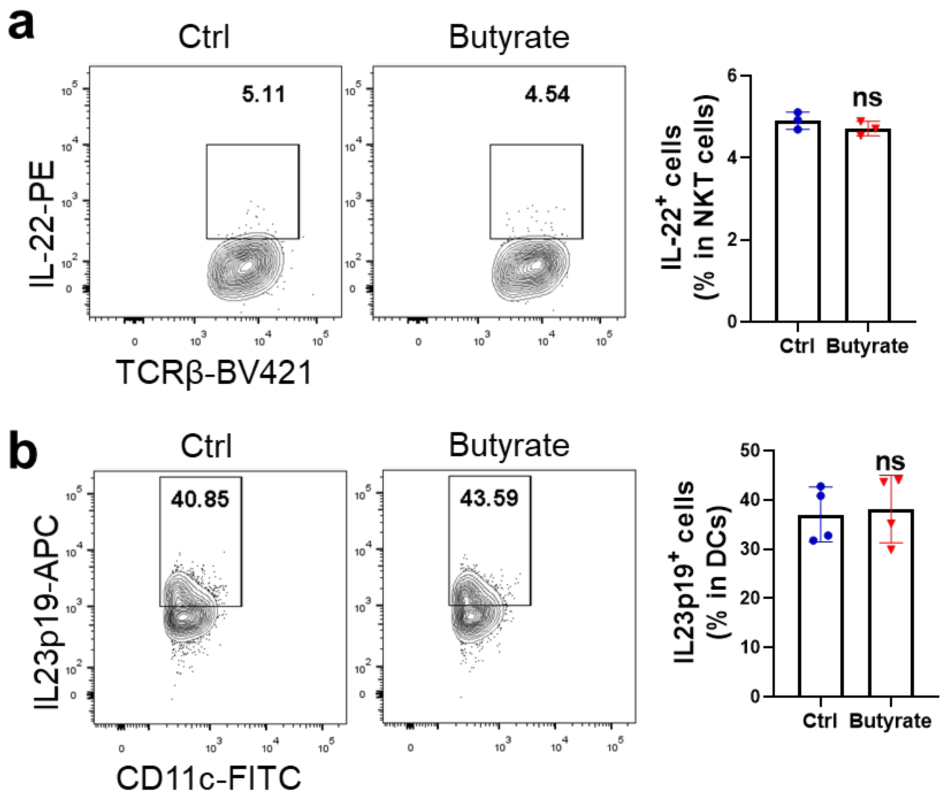

Supplementary Fig. 5. Butyrate does not affect IL-22 production in NKT cells and IL-23 production in dendritic cells. (**a**) Intestinal lamina propria cells were treated with IL-23 (20 ng/ml) in the presence or absence of butyrate (0.5 mM) for 16 h, and IL-22 production was measured by gating on NK1.1^{+} cells by flow cytometry. (**b**) WT mice were treated with or without 200 mM butyrate in drinking water for 3 weeks (n = 4/group). IL-23p19 levels in intestinal dendritic cells (DCs) were measured by flow cytometry. One representative of three independent experiments was shown. The data was expressed as mean ± SD. Statistical significance was tested by two-tailed unpaired Student *t* test.

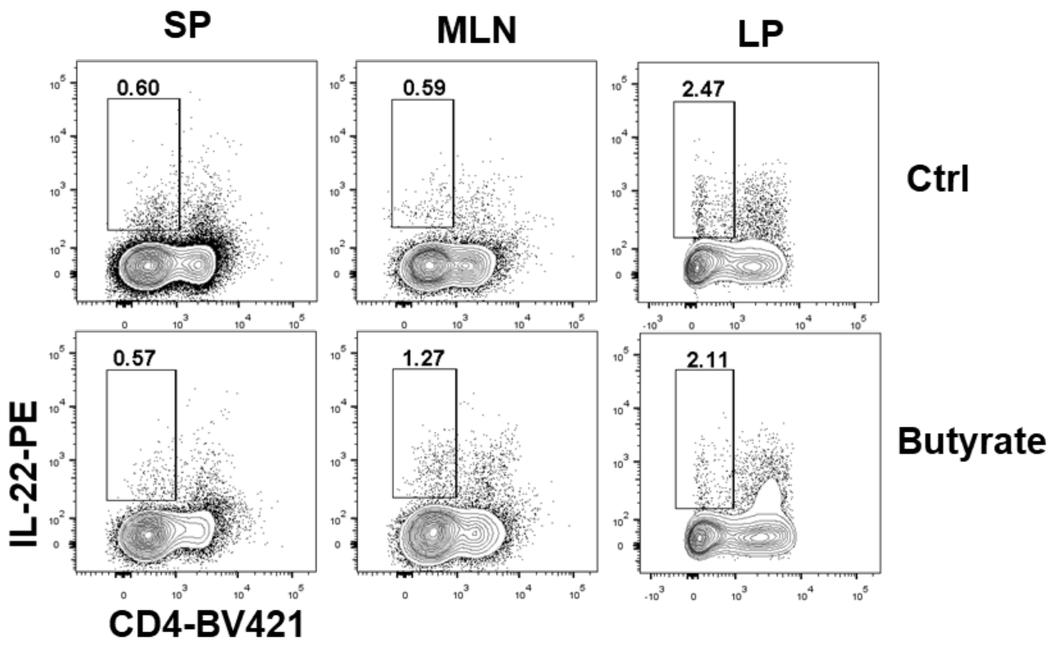

Supplementary Fig. 6. CD4⁻ cells produce IL-22 in spleen, MLN, and intestinal lamia propria. WT mice were treated with or without 200 mM butyrate in drinking water for 3 weeks (n = 4/group). Mice were sacrificed on day 21, and IL-22-producing cells in live cells was measured by flow cytometry One representative of three independent experiments was shown.

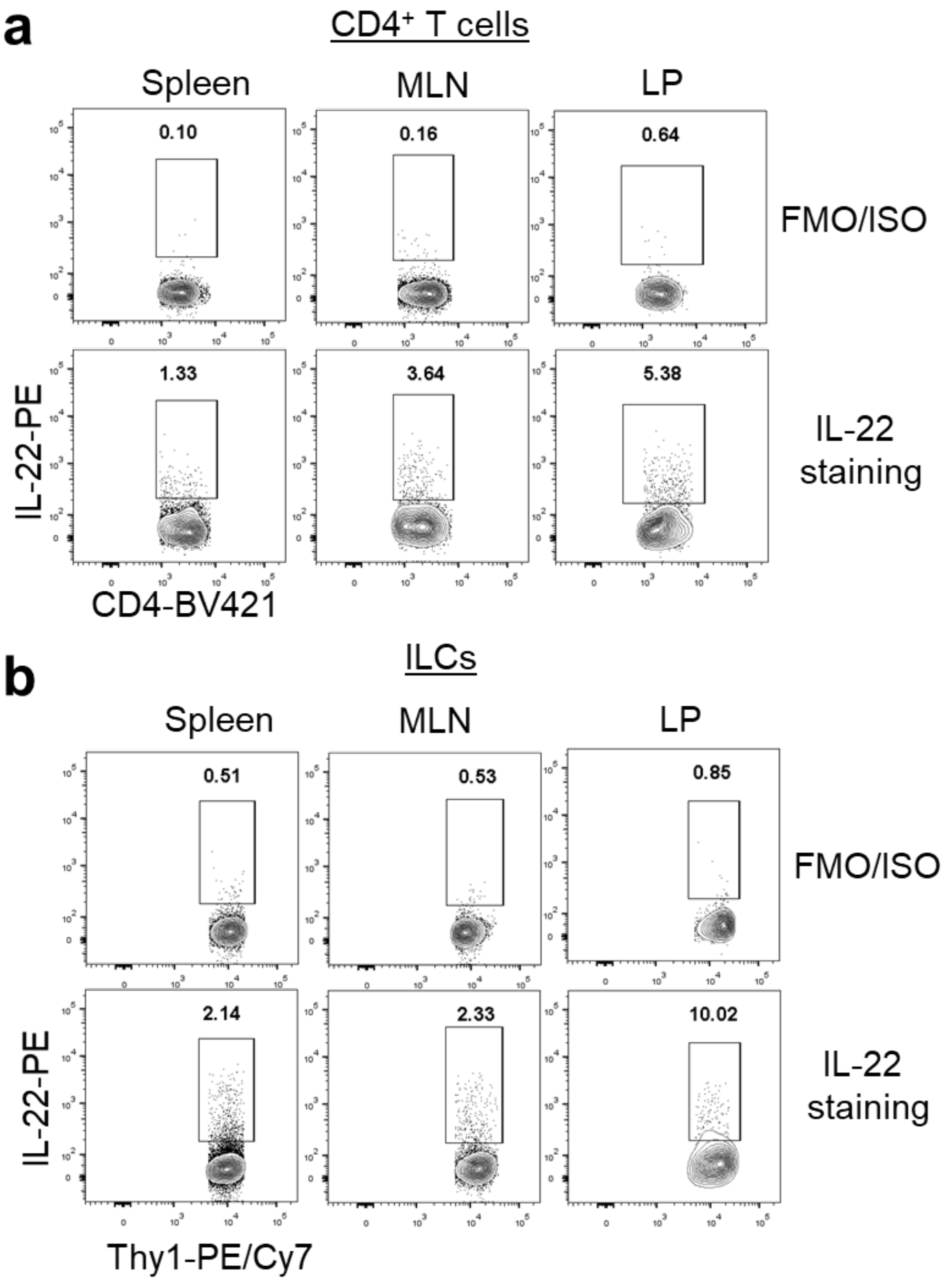

Figure S7. Fluorescence Minus One/Isotype (FM/ISO) staining. (**a**) Cells were isolated from spleen, MLN, and LP, and stimulated with ionomycin and PMA for 2 h, then brefeldin was added for another 3 h. Cells were first incubated with anti-CD16/32 for Fc blocking, and then stained

with Live/dye-APC/Cy7, followed by surface staining with BV421 anti-mouse CD4 antibody. After fixation, cells were stained with anti-IgG Isotype (FMO/ISO) or PE anti-IL-22 (IL-22 staining) antibody. The percentages of IL-22 were assessed by flow cytometry. (**b**) Cells were isolated from spleen, MLN, and LP were stimulated with IL-23 for 16 h, and then activated with ionomycin and PMA for 2 h, followed by adding brefeldin for another 3 h. Cells were Cells were first incubated with anti-CD16/32 for Fc blocking, and then stained with Live/dye-APC/Cy7, followed by surface staining with PE/Cy7-anti Thy1 and FITC-lineage (CD3, CD11b, CD11c, B220, NK1.1, and Gr1) antibodies. After fixation, cells were stained with anti-IgG Isotype (FMO/ISO) or PE anti-IL-22 (IL-22 staining) antibody. The percentages of IL-22 were assessed by flow cytometry.

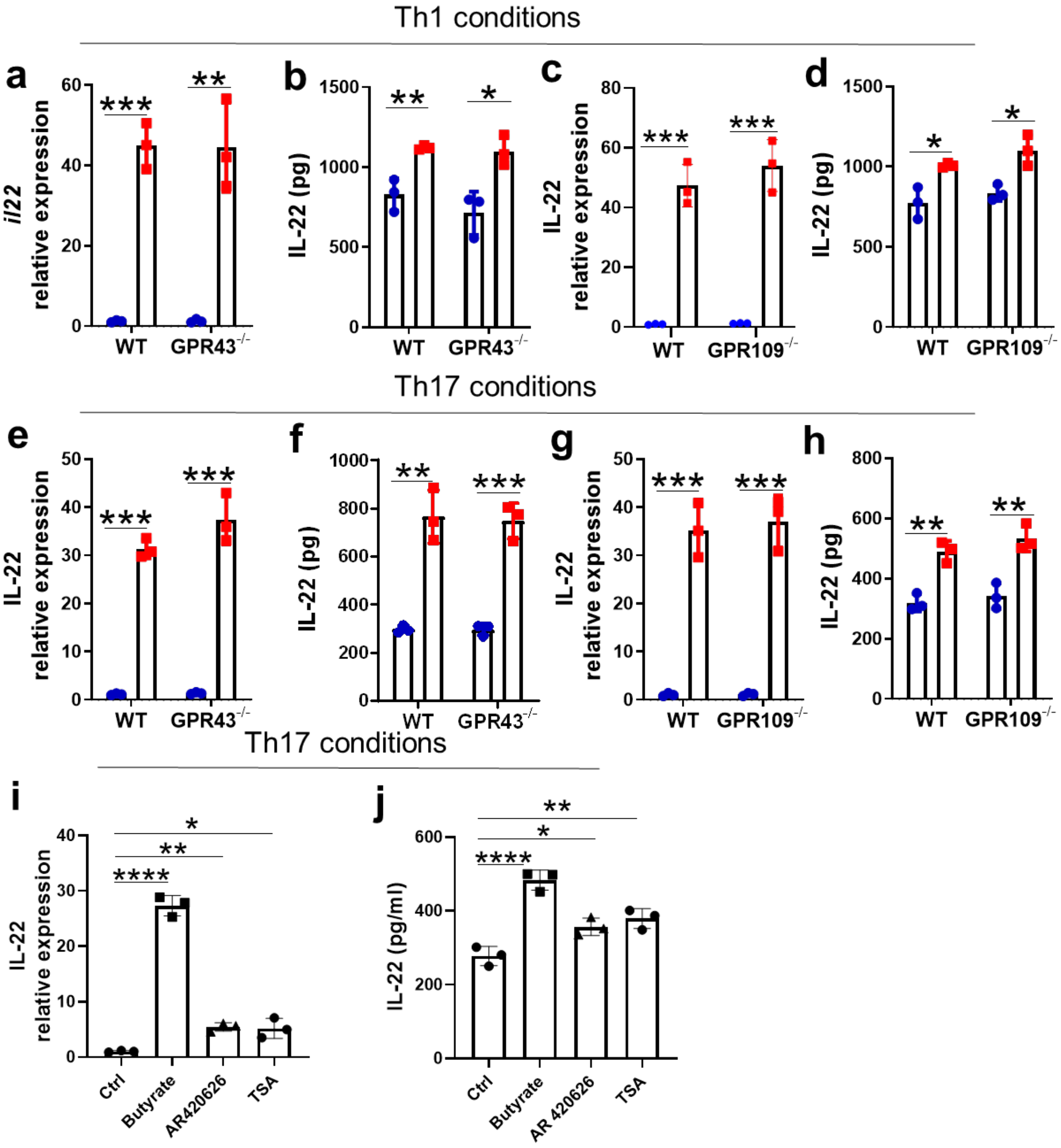

Supplementary Fig. 8. Butyrate upregulates IL-22 production in CD4$^+$ T cells through GPR41 and inhibiting HDAC, but not GPR43 and GPR109a. (a-h) WT, GPR43$^{-/-}$, and GPR109a$^{-/-}$ CD4$^+$ T cells were activated with anti-CD3 and anti-CD28 mAb under Th1 (**a-d**) or Th17 (**e-h**) conditions with or without butyrate (0.5 mM) (n=3/group). I*l22* expression was analyzed by using qRT-PCR (**a, c, e, and g**) and ELISA (**b, d, f, and h**). (**i-j**) CBir1 Tg CD4$^+$ T

cells were activated with APCs and 1 μg/ml CBir1 peptide with or without butyrate (0.5 mM) ± AR420626 (5 μM) or TSA (10 mM) under Th17 conditions for 60 h (n=3/group). IL-22 mRNA (**i**) and protein (**j**) were measured by qRT-PCR and ELISA. One representative of three independent experiments was shown. The data was expressed as mean ± SD. Statistical significance was tested by two-tailed unpaired Student *t* test (**a-h**) or two-tailed one-way ANOVA (**i-j**). **a**, ***p = 0.0002, **p = 0.0025; **b**, **p = 0.0075, *p = 0.0153; **c**, ***p = 0.0003 (WT) and 0.0005 ($GPR109^{-/-}$); **d**, *p = 0.0156 (WT) and 0.0137 ($GPR109^{-/-}$); **e**, ****p < 0.0001, ***p = 0.0003; **f**, **p = 0.0019, ***p = 0.0005; **g**, ***p = 0.0005 (WT) and 0.0003 ($GPR109^{-/-}$); **h**, **p = 0.0029 (WT) and 0.0054 ($GPR109^{-/-}$); **i**, ****p < 0.0001, **p = 0.0097, *p = 0.0133; **j**, ****p < 0.0001, *p = 0.0145, **p = 0.0036.

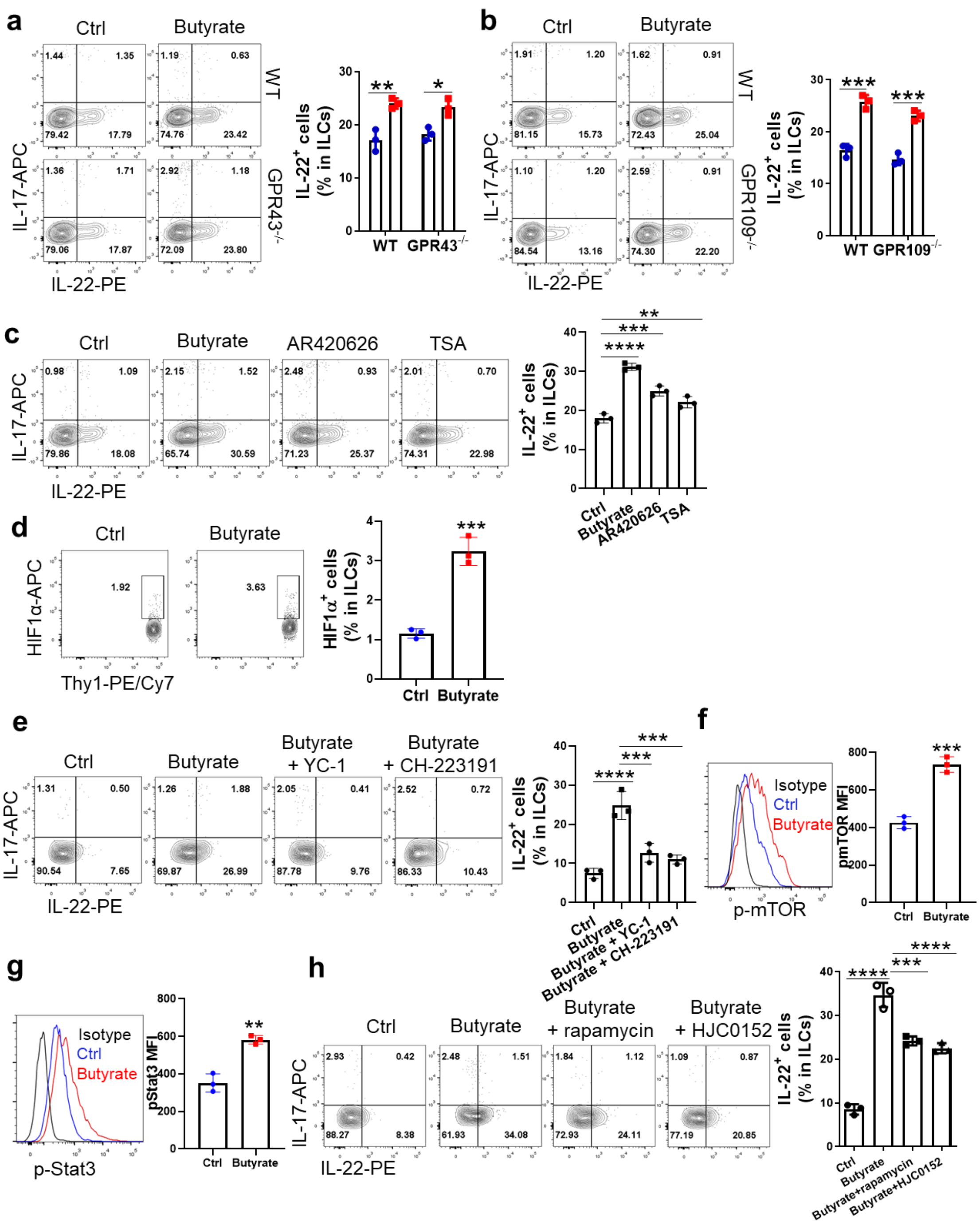

Supplementary Fig. 9. Butyrate promotes IL-22 production in ILCs. *(***a-b**) CD4+ T cell-depleted splenic cells from WT, *Gpr43*$^{-/-}$ (**a**), or *Gpr109a*$^{-/-}$ mice (**b**) were stimulated with IL-23 (20 ng/ml) in the presence or absence of butyrate (0.5 mM) for 16 h (n=3/group). IL-22 production

in ILCs were analyzed by flow cytometry. (**c**) $CD4^+$ T cell-depleted splenic cells were stimulated with IL-23 (20 ng/ml) with or without butyrate (0.5 mM) in the presence or absence of AR420626 (5 μM) or TSA (10 mM) for 16 h (n = 3/group). IL-22 production in ILCs was analyzed by flow cytometry. (**d**) $CD4^+$ T cell-depleted splenic cells were stimulated with IL-23 (20 ng/ml) in the presence or absence of butyrate (0.5 mM) for 16 h (n = 3/group). HIF1α in ILCs ($Lineage^-$ $Thy1^+$) were analyzed by flow cytometry. (**e**) $CD4^+$ T cell-depleted splenic cells were stimulated with IL-23 (20 ng/ml) with or without butyrate (0.5 mM) in the presence or absence of YC-1 (5 μM) or CH-223191 (3 μM) for 16 h (n = 3/group). IL-22 production in ILCs were analyzed by flow cytometry. (**f-g**) $CD4^+$ T cell-depleted splenic cells were stimulated with IL-23 (20 ng/ml) in the presence or absence of butyrate (0.5 mM) for 2 h (n=3/group). pmTOR (**f**) and pStat3 (**g**) levels were measured by flow cytometry. (**h**) $CD4^+$ T cell-depleted splenic cells were stimulated with IL-23 (20 ng/ml) with or without butyrate (0.5 mM) in the presence or absence of rapamycin (1 μM) or HJC0152 (1 μM) for 16 h (n=3/group). IL-22 production in ILCs was analyzed by flow cytometry. The data was expressed as mean ± SD. The data was expressed as mean ± SD. Statistical significance was tested by two-tailed unpaired Student *t* test (**a-b, d, and f-g**) or two-tailed one-way ANOVA (**c, e, and h**). **a**, $^{**}p = 0.0061$, $^{*}p = 0.0129$; **b**, $^{***}p = 0.0009$ (WT) and 0.0006 ($GPR109^{-/-}$); **c**, $^{****}p < 0.0001$, $^{***}p = 0,0003$, $^{**}p = 0.0082$; **d**, $^{***}p = 0.0006$; **e**, $^{****}p < 0.0001$, $^{***}p = 0.0006$ (butyrate + YC-1 vs butyrate) and 0.0002 (butyrate + CH-223191 vs butyrate); **f**, $^{***}p = 0.0005$; **g**, $^{**}p = 0.0018$; **h**, $^{****}p < 0.0001$, $^{***}p = 0.0002$.

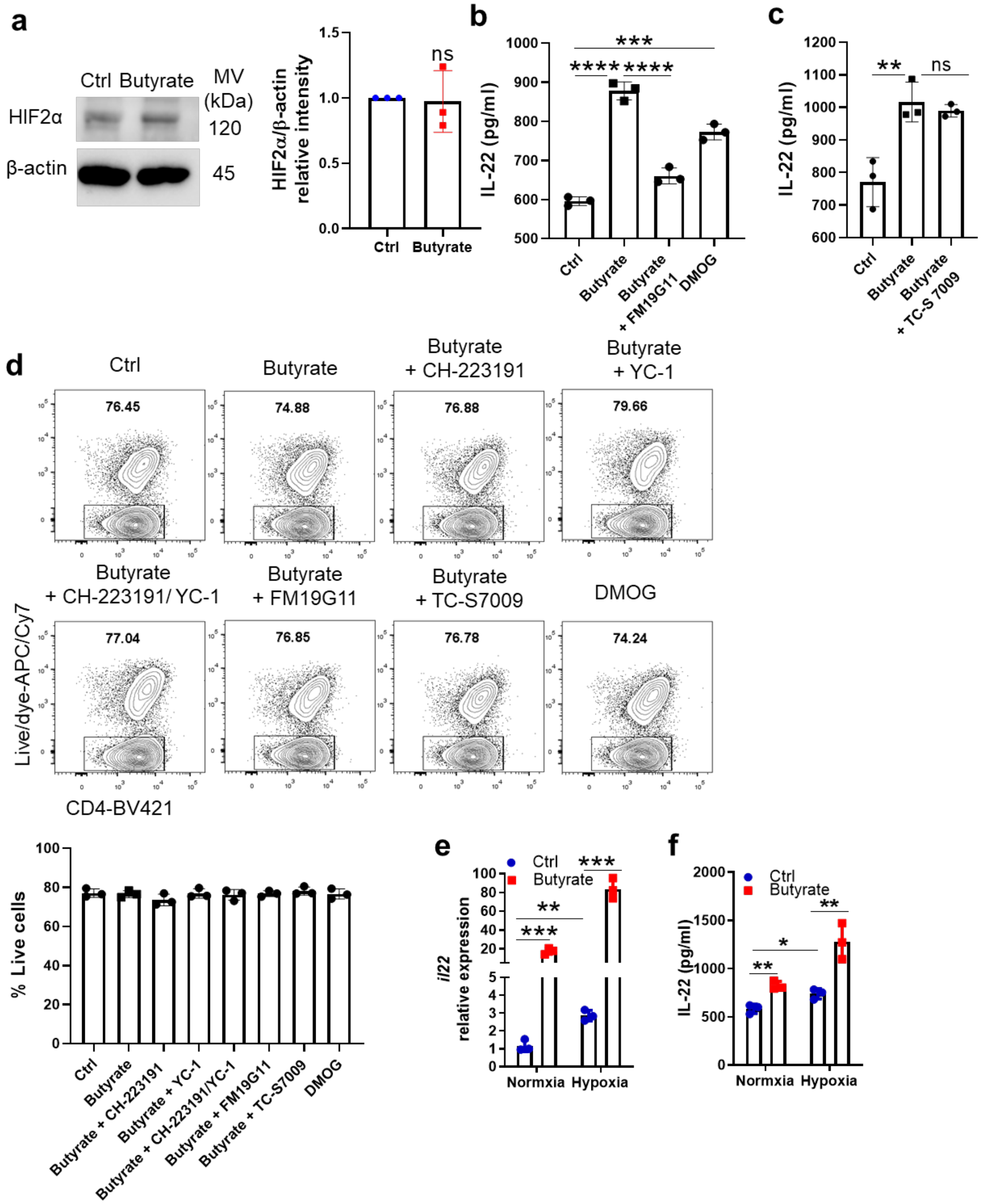

Supplementary Fig. 10. Butyrate promotes IL-22 production through HIF1α but not HIF2α.

(**a**) WT CD4^{+} T cells were activated with anti-CD3 and anti-CD28 mAb under Th1 conditions with or without butyrate (0.5 mM) for 2 days (n=3/group), and HIF2α protein was analyzed by

western blot (ns, no significance). (**b**) CBir1 Tg $CD4^+T$ cells were activated with APCs and 1 μg/ml CBir1 peptide under Th1 condition with butyrate (0.5 mM) ± FM19G11 (500 nM), or DMOG (100 μM) for 60 h (n=3/group). IL-22 expression was measured by ELISA. (**c**) CBir1 Tg $CD4^+T$ cells were activated with APCs and 1 μg/ml CBir1 peptide under Th1 conditions with butyrate (0.5 mM) ± TC-S 7009 (50 μM) for 60 h (n=3/group). IL-22 expression was measured by ELISA. (**d**) CBir1 Tg $CD4^+T$ cells were activated with APCs and 1 μg/ml CBir1 peptide under Th1 conditions with butyrate (0.5 mM) in the presence of different inhibitor and agonist for 5 days, and cell viability was assessed by live cells using Live/dye staining. (**e-f**) $CD4^+$ T cells were activated with anti-CD3 and anti-CD28 mAb treated under Th1 conditions with or without butyrate (0.5 mM) under normoxic or hypoxic (3 % O_2) conditions for 60 h (n = 3/group). IL-22 mRNA (**e**) and protein (**f**) were measured by qRT-PCR and ELISA. One representative of three independent experiments was shown. The data was expressed as mean ± SD. Statistical significance was tested by two-tailed unpaired Student *t* test (**a, e, and f**) or two-tailed one-way ANOVA (**b-d**). **b**, *****p* < 0.001, ****p* = 0.0004; **c**, ***p* = 0.0033, ns, no significance; **e**, ****p* = 0.0001 (normoxia) and 0.0002 (hypoxia), ***p* = 0.0027; **f**, ***p* = 0.0032 (normoxia) and 0.0085 (hypoxia), **p* = 0.0193.

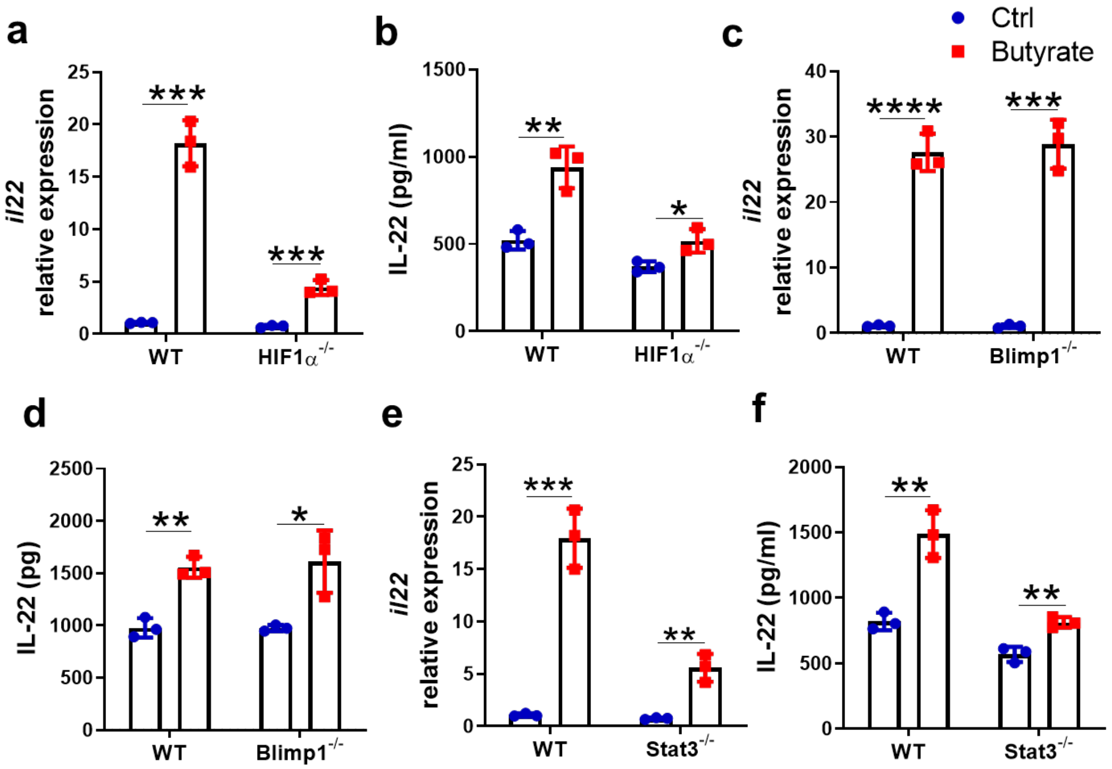

Supplementary Fig. 11. Butyrate promotes CD4^{+} T cell IL-22 production through HIF1α and Stat3, but not Blimp1. WT, HIF1α-deficient, Blimp1-deficient, and Stat3-deficient CD4^{+} T cells were activated with anti-CD3 and anti-CD28 mAb under Th1 condition with or without butyrate (0.5 mM) (n=3/group). IL-22 expression was analyzed by using qRT-PCR (**a, c, and e**) and ELISA (**b, d, and f**). One representative of three independent experiments was shown. The data was expressed as mean ± SD. Statistical significance was tested by two-tailed unpaired Student *t* test. **a**, ***$p = 0.0002$ and 0.0008; **b**, **$p = 0.0052$, *$p = 0.0256$; **c**, ****$p < 0.0001$, ***$p = 0.0002$; **d**, **$p = 0.0018$, *$p = 0.0211$; **e**, ***$p = 0.0005$, **$p = 0.0032$; **f**, **$p = 0.0039$.

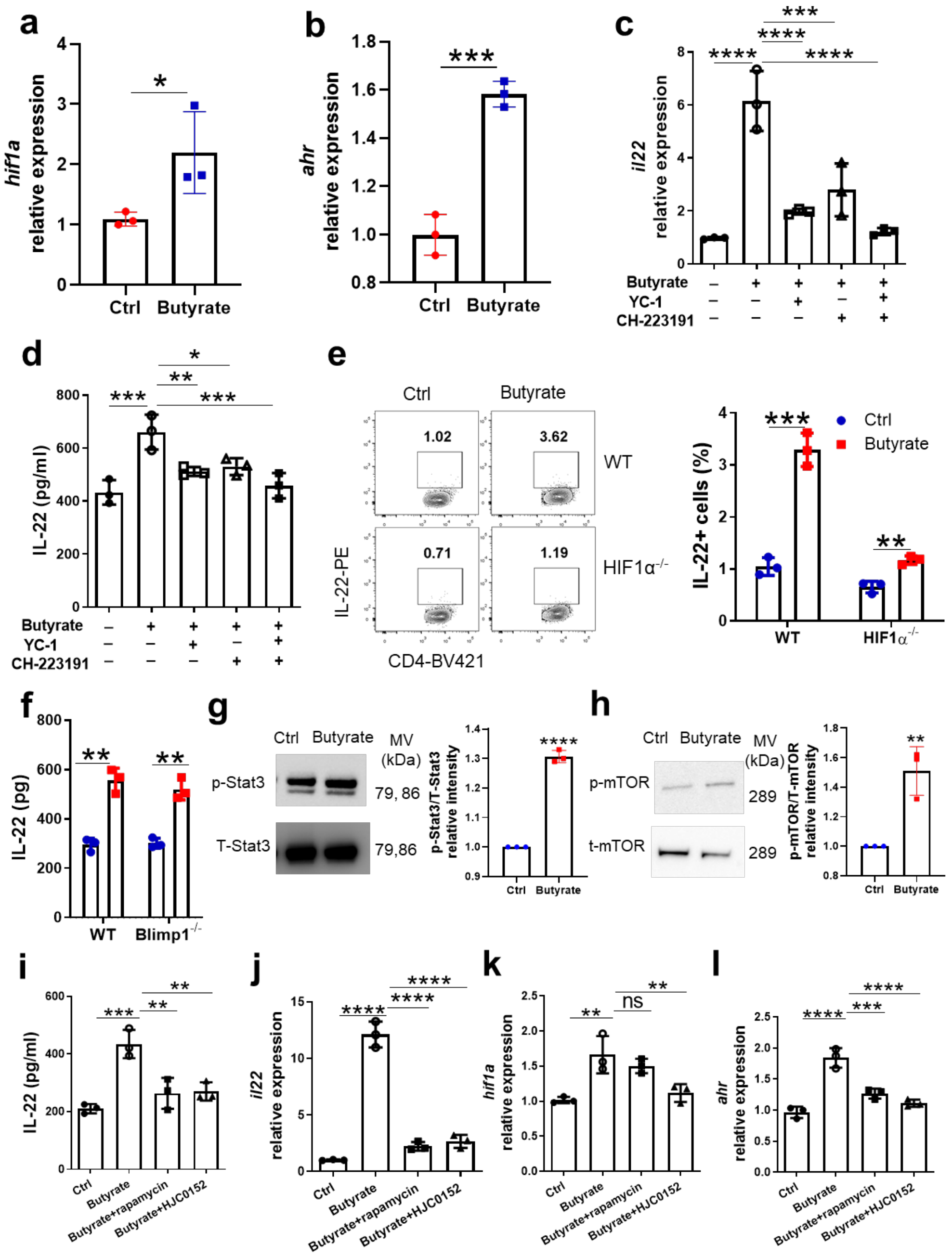

Supplementary Fig. 12. Butyrate induces HIF1α and AhR to promote IL-22 expression through mTOR and Stat3 pathways under Th17 conditions. (**A-B**) CBir1 CD4^{+} T cells were activated with APCs and 1 μg/ml Cbir1 peptide under Th17 conditions with or without butyrate (0.5 mM) (n=3/group). Expression of H*if1a* (**a**) and A*hr* (**b**) was analyzed by using qRT-PCR. (**c-**

d) CBir1 CD4$^+$ T cells were activated with APCs and 1 μg/ml Cbir1 peptide under Th17 conditions with butyrate (0.5 mM) in the presence or absence of YC-1 (5 μM) or/and CH-223191 (3 μM) for 60 h (n=3/group). IL-22 mRNA (**c**) and protein (**d**) levels were measured by qRT-PCR and ELISA. (**e**) WT and HIF-1α-deficient CD4$^+$ T cells were activated with anti-CD3 and anti-CD28 mAb in the presence or absence of butyrate (0.5 mM) for 5 days (n = 3/group). IL-22 production was assessed by flow cytometry. (**f**) WT and Blimp1-deficient CD4$^+$ T cells were activated with anti-CD3 and anti-CD28 under Th17 condition in the presence or absence of butyrate (0.5 mM) (n=3/group). IL-22 production was analyzed by using ELISA at 60 h. (**g-h**) WT splenic CD4$^+$ T cells were activated with anti-CD3 and anti-CD28 mAb under Th17 condition in the presence or absence of butyrate (0.5 mM). Phosphorylated Stat3 was analyzed by western blot at 12 h (**g**), and phosphorylated mTOR at 24 h (**h**). (**i-l**) CBir1 CD4$^+$ T cells were activated with APCs and 1 μg/ml Cbir1 peptide under Th17 condition with butyrate (0.5 mM) in the presence or absence of rapamycin (1 μM) or HJC0152 (1 μM) (n = 3/group). IL-22 mRNA (**i**) and protein (**j**) levels were assessed by qRT-PCR and ELISA at 60 h. Expression of H*if1a* (**k**) and A*hr* (**l**) was analyzed at 2 days by qRT-PCR. One representative of three independent experiments was shown. The data was expressed as mean ± SD. Statistical significance was tested by two-tailed unpaired Student *t* test (**a-b, and e-h**) or two-tailed one-way ANOVA (**c-d, and i-l**). **a**, *$p = 0.0499$; **b**, ***$p = 0.0005$; **c**, ****$p < 0.0001$, ***$p = 0.0004$; **d**, ***$p = 0.0004$ (butyrate vs control) and 0.0009 (butyrate + YC-1 + CH-223191 vs butyrate; **e**, (***$p = 0.0004$, **$p = 0.0029$); **f**, *$p = 0.0012$ (WT) and 0.0013 (Blimp1$^{-/-}$); **g**, ****$p < 0.0001$; **h**, **$p = 0.0059$; **i**, ***$p = 0.0004$, **$p = 0.0022$ (butyrate + rapamycin vs butyrate) and 0.0028 (butyrate + HJC0152 vs butyrate); **j**, ****$p < 0.0001$; **k**, **p = 0.0074 (butyrate vs control) and 0.0027 (butyrate + HJC0152 vs butyrate), ns, no significance; **l**, ****$p < 0.0001$, ***$p = 0.0004$;

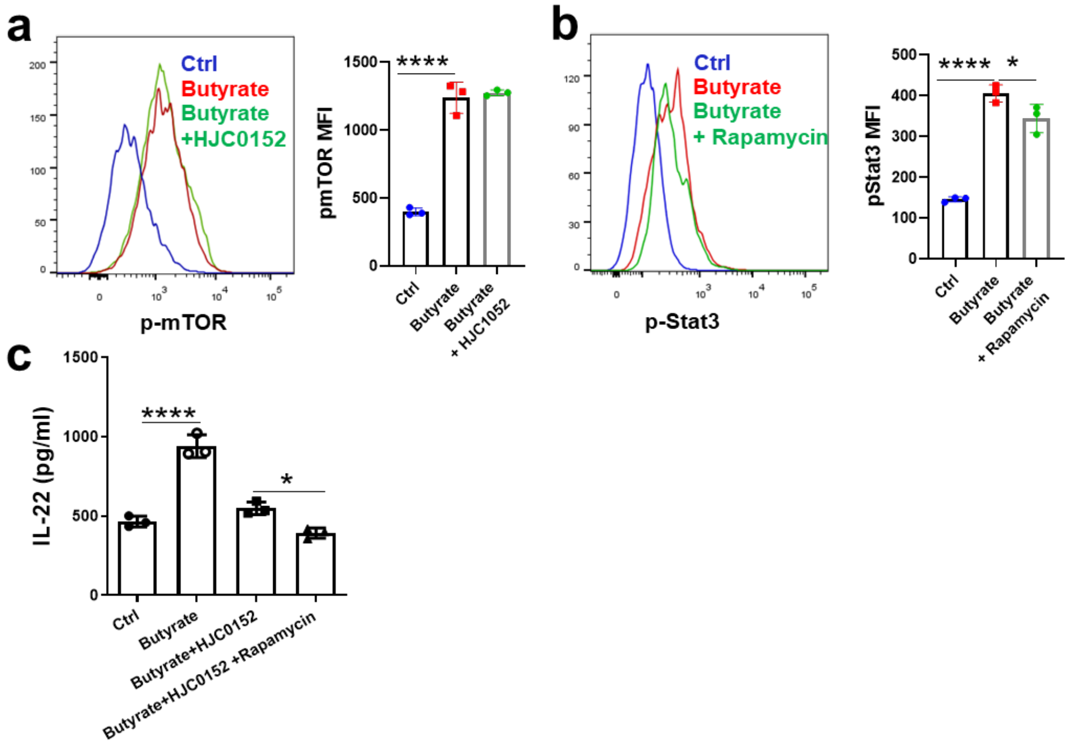

Supplementary Fig. 13. Rapamycin moderately suppresses Stat3 activation induced by butyrate. (**a**) WT CD4$^+$ T cells were activated with anti-CD3 and anti-CD28 mAb under Th1 conditions with or without butyrate (0.5 mM) in the presence or absence of HJC0152 (1 μM), and phosphorylated mTOR (24 h) were assessed by flow cytometry. (**b**) CD4$^+$ T cells were activated with anti-CD3 and anti-CD28 mAb under Th1 conditions with or without butyrate (0.5 mM) in the presence or absence of rapamycin (1 μM), and Phosphorylated Stat3 (6 h) were assessed by flow cytometry. (**c**) CD4$^+$ T cells were activated under Th1 conditions with butyrate (0.5 mM) in the presence or absence of HJC0152 (1 μM) ± rapamycin (1 μM) (n=3/group). IL-22 levels were assessed by ELISA at 60 h. One representative of three independent experiments was shown. The data was expressed as mean ± SD. Statistical significance was tested by two-tailed one-way ANOVA. **a**, ****$p < 0.0001$; **b**, ****$p < 0.0001$, *$p = 0.0332$; **c**, ****$p < 0.0001$, *$p = 0.0148$.

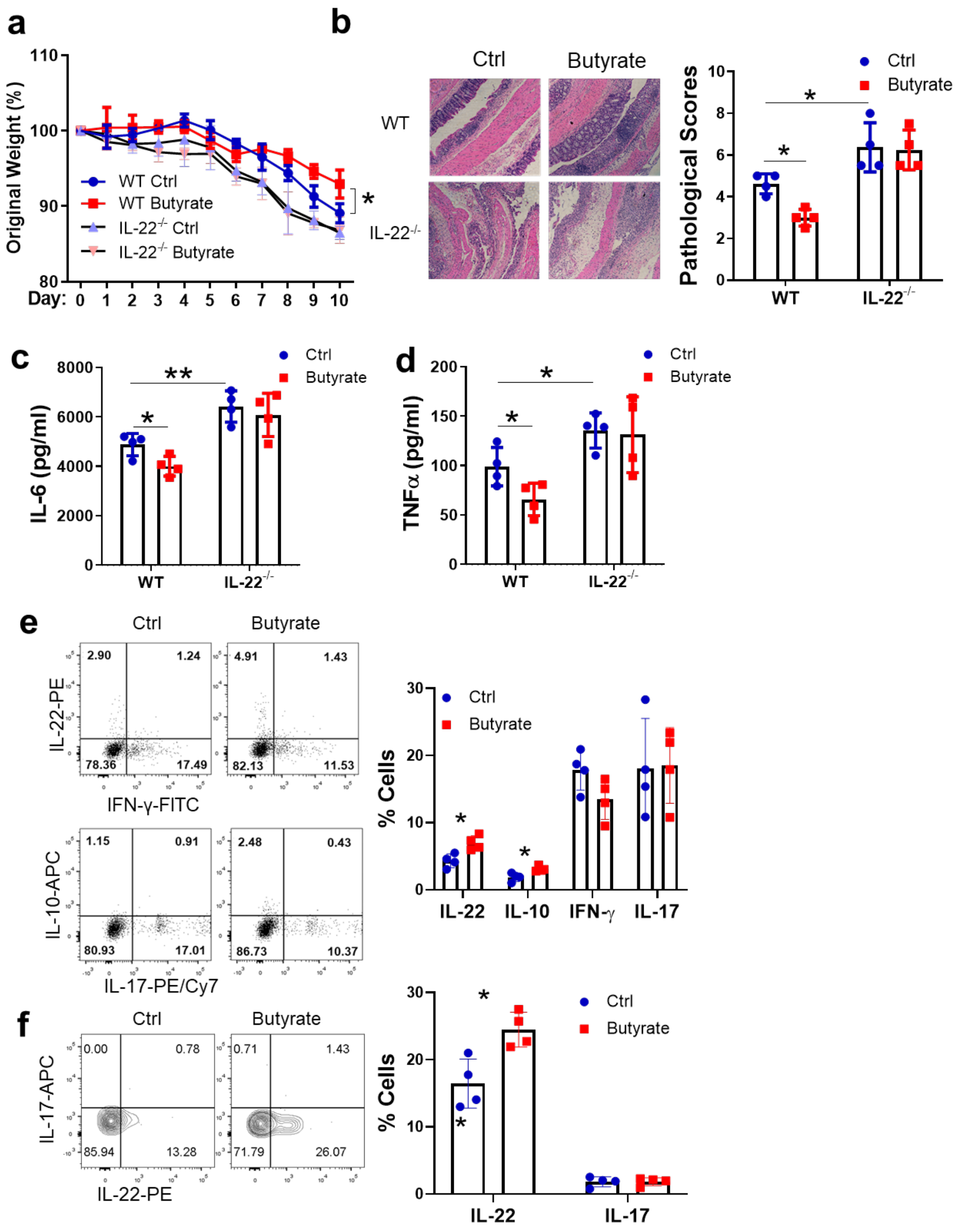

Supplementary Fig. 14. Butyrate ameliorates DSS-induced colitis through promoting IL-22 production. WT and *Il22*$^{-/-}$ mice were administrated with DSS in the presence or absence of butyrate (200 mM) in drinking water for 7 days, followed by drinking water with or without

butyrate for another 3 days (n=4 mice/group). Mice were weighed daily (**a**), and colonic histopathology and pathology score (**b**), colonic IL-6 (**c**), and TNF-α (**d**) production in colonic organ culture were determined at day 10. IL-22^{+}, IL-10^{+}, IFN-γ^{+}, and IL-17^{+} CD4^{+} T cells (**e**), and IL-22^{+} and IL-17^{+} ILCs (Lineage^{-} Thy1^{+}) (**f**) (**p* = 0.0116) in LP of WT mice were determined at day 10. One representative of three independent experiments was shown. The data was expressed as mean ± SD. Statistical significance was tested by two-tailed unpaired Student *t* test (**a, c-f**) or the nonparametric two-tailed Mann-Whitney U test (**b**). **a**, **p* = 0.0139; **b**, **p* = 0.0286; **c**, **p* = 0.0280, ***p* = 0.0075; **d**, **p* = 0.0400 (WT butyrate vs WT control) and 0.0322 (IL-22$^{-/-}$ control vs WT control); **e**, **p* = 0.0115 (IL-22) and 0.0170 (IL-10).

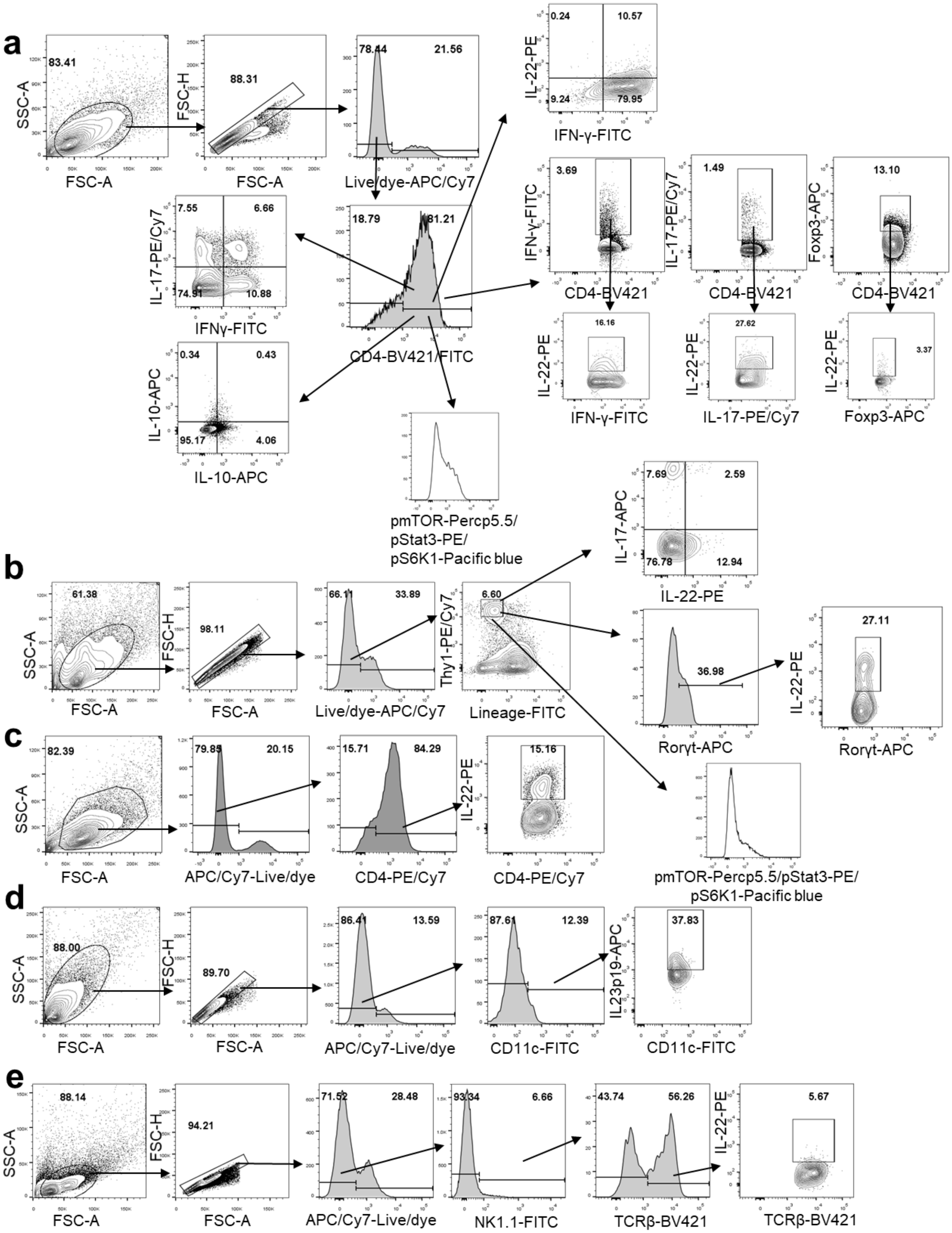
a
SSC-A
FSC-A
83.41
FSC-H
88.31
78.44
21.56
Live/dye-APC/Cy7
IL-22-PE
IFN-γ-FITC
0.24
10.57
9.24
79.95
IL-17-PE/Cy7
IFNγ-FITC
7.55
6.66
74.91
10.88
CD4-BV421/FITC
18.79
81.21
IFN-γ-FITC
CD4-BV421
3.69
IL-17-PE/Cy7
1.49
Foxp3-APC
13.10
16.16
27.62
3.37
IL-17-PE/Cy7
Foxp3-APC
IL-10-APC
0.34
0.43
95.17
4.06
pmTOR-Percp5.5/
pStat3-PE/
pS6K1-Pacific blue
b
61.38
98.11
66.11
33.89
Thy1-PE/Cy7
Lineage-FITC
6.60
IL-17-APC
IL-22-PE
7.69
2.59
76.78
12.94
Rorγt-APC
36.98
27.11
c
82.39
APC/Cy7-Live/dye
79.85
20.15
CD4-PE/Cy7
15.71
84.29
15.16
pmTOR-Percp5.5/pStat3-PE/
pS6K1-Pacific blue
d
88.00
89.70
86.41
13.59
CD11c-FITC
87.61
12.39
IL23p19-APC
37.83
e
88.14
94.21
71.52
28.48
NK1.1-FITC
93.34
6.66
TCRβ-BV421
43.74
56.26
5.67

Supplementary Fig. 15. Flow cytometry gating strategies. (**a**) Gating strategies for mouse $CD4^+$ T cells used in Fig. 1h, 2e, 2f, 3c, 4j-k, 5b, 5d, 5e-j, 7c, Supplementary Fig. 1j, 2, 3a-c, 6, 7a, 10d, 12e, 13a-b, and 14e. (**b**) Gating strategies for ILCs used in Fig. 1i, 2g, 2h, 7d, Supplementary Fig. 3d, 4, 7b, 9, and 14f. (**c**) Gating strategies for human $CD4^+$ T cells used in Fig. 9b and 9f. (**d**) Gating strategies for mouse DCs used in Supplementary Fig. 5b. (**e**) Gating strategies for mouse NKT cells used in Supplementary Fig. 5B.

Supplementary Table 1. Clinical characteristics of patients with active Crohn's diseases (CD), Ulcerative colitis (UC) and healthy donors.

	HC	CD	UC
**Number**	8	10	7
**Age (years)**	27.03±6.27	28.91±10.18	38.13±13.66
**Gender**			
Male	4	6	4
Female	4	4	3
**Disease duration (months)**	N/A	41.32±15.37	46.12±12.66
**Current therapy**			
5-aminosalicylates	N/A	3	2
Immunosuppressants	N/A	0	N/A
Biologics	N/A	0	N/A
Nutritional therapy	N/A	6	4
**Disease location (CD)#**			
L1	N/A	3	N/A
L2	N/A	3	N/A
L3	N/A	4	N/A
L4	N/A	0	N/A
**Disease location (UC)#**			
E1	N/A	N/A	2
E2	N/A	N/A	5
E3	N/A	N/A	0

#According to the Montreal classification system.

Supplementary Table 2. Primers for qRT-PCR and CHIP assay

**Primers for qRT-PCR**			
**Genes**	**Species**	**Forward Primer**	**Reverse Primer**
Gapdh	Mouse	GGTTGTCTCCTGCGACTTCA	TGGTCCAGGGTTTCTTACTCC
Iil22	Mouse	AACAGCAGGTCCAGTTCCCC	GCTCCTGTCACATCAGCGGT
Aahr	Mouse	TTGGTTGTGATGCCAAAGGGC	CATGCGGATGTGGGATTCTGC
Hif1a	Mouse	AGCTTCTGTTATGAGGCTCACC	TGACTTGATGTTCATCGTCCTC
Tbx21	Mouse	AGCAAGGACGGCGAATGTT	GGGTGGACATATAAGCGGTTC
Gapdh	Human	CAGGAGGCATTGCTGATGAT	GAAGGCTGGGGCTCATTT
Il22	Human	TGCTGTTCCCTCAATCTG	TGTGCTTAGCCTGTTGCTG
Ahr	Human	CGTCTAAGGTGTCTGCTGGAT	CAAGTATGGATGGTGGCTGAAG
Hif1a	Human	ACTAGCCGAGGAAGAACTATGA	CTGAGGTTGGTTACTGTTGGTA
**Primers for CHIP**			
**Name**	**Species**	**Forward Primer**	**Reverse Primer**
Il22 (HRE)	Mouse	GGGAGAAAGATCTCACACAGT	CAGACGCCTACCCTAGACT
