# Supplementary material for: Intestinal microbiota-derived short-chain fatty acids regulation of immune cell IL-22 production and gut immunity
Source: Nat Commun. 2020 Sep 8;11:4457. doi: 10.1038/s41467-020-18262-6 (PMC7478978; doi:10.1038/s41467-020-18262-6)
Supplement: Supplementary file 3 — Reporting Summary [file 41467_2020_18262_MOESM3_ESM.pdf]

nature research

Corresponding author(s): Yingzi Cong

Last updated by author(s): 05/30/2020

Reporting Summary

Nature Research wishes to improve the reproducibility of the work that we publish. This form provides structure for consistency and transparency in reporting. For further information on Nature Research policies, seeAuthors & Referees and theEditorial Policy Checklist .

Statistics

For all statistical analyses, confirm that the following items are present in the figure legend, table legend, main text, or Methods section.

n/a	Confirmed	
[ ]	[x]	The exact sample size (*n*) for each experimental group/condition, given as a discrete number and unit of measurement
[ ]	[x]	A statement on whether measurements were taken from distinct samples or whether the same sample was measured repeatedly
[ ]	[x]	The statistical test(s) used AND whether they are one- or two-sided<br>*Only common tests should be described solely by name; describe more complex techniques in the Methods section.*
[x]	[ ]	A description of all covariates tested
[x]	[ ]	A description of any assumptions or corrections, such as tests of normality and adjustment for multiple comparisons
[ ]	[x]	A full description of the statistical parameters including central tendency (e.g. means) or other basic estimates (e.g. regression coefficient) AND variation (e.g. standard deviation) or associated estimates of uncertainty (e.g. confidence intervals)
[ ]	[x]	For null hypothesis testing, the test statistic (e.g. *F*, *t*, *r*) with confidence intervals, effect sizes, degrees of freedom and *P* value noted<br>*Give P values as exact values whenever suitable.*
[x]	[ ]	For Bayesian analysis, information on the choice of priors and Markov chain Monte Carlo settings
[x]	[ ]	For hierarchical and complex designs, identification of the appropriate level for tests and full reporting of outcomes
[x]	[ ]	Estimates of effect sizes (e.g. Cohen's *d*, Pearson's *r*), indicating how they were calculated

Our web collection on statistics for biologists contains articles on many of the points above.

Software and code

Policy information about availability of computer code

Data collection	BD FACS Diva software 8.0.1
Data analysis	Graphpad Prism8, Flowjo 10.0, R3.4.0, Novosmart 1.0

For manuscripts utilizing custom algorithms or software that are central to the research but not yet described in published literature, software must be made available to editors/reviewers. We strongly encourage code deposition in a community repository (e.g. GitHub). See the Nature Research guidelines for submitting code & software for further information.

Data

Policy information about availability of data

All manuscripts must include a data availability statement. This statement should provide the following information, where applicable:

- Accession codes, unique identifiers, or web links for publicly available datasets
- A list of figures that have associated raw data
- A description of any restrictions on data availability

All data supporting the findings of this study are available within the article and its supplementary information files or from the corresponding author upon reasonable request. RNA seq data has been deposited in GEO database under the accession number GSE139631. The source data underlying Fig 1a-i, 2a-h, 3a-d, 4a-m, 5a-j, 6b-e, 7a-h, 8a-f, 9a-f, and Supplementary 1c-e, 1h-j, 2, 3a, 3d, 4, 5a-b, 8a-j, 9a-h, 10a-f, 11a-f, 12a-l, 13a-c, S14a-f. Source data were provided as a Source Data file.

Field-specific reporting

Please select the one below that is the best fit for your research. If you are not sure, read the appropriate sections before making your selection.

Life sciences study design

All studies must disclose on these points even when the disclosure is negative.

Sample size	Sample size were determined based on the experimental setting. For mouse models, the number of mice in each group was calculated in order to demonstrate a statistically significance difference at p less than 0.5 and power no less than 80%. For in vitro experiments, sample size were determined by magnitude and consistency of measurable differences. The precise number of cell samples, animals used were indicated in the figure legends.
Data exclusions	No data were excluded from analysis in this study.
Replication	Experiments were repeated. Our data represent at least two to three independent experiments with similar results.
Randomization	For animal study, all the animals were randomly divided into different group for experiments. For human samples, patients and control samples were randomly collected.
Blinding	Investigators were blinded for disease scoring. The investigators were not blinded for cell harvest or processing due to the risk of confusion in handling. The investigators were not blinded during acquisition or analysis of flow cytometry data, but the same gating were applied to all samples in the given experiment.

Reporting for specific materials, systems and methods

We require information from authors about some types of materials, experimental systems and methods used in many studies. Here, indicate whether each material, system or method listed is relevant to your study. If you are not sure if a list item applies to your research, read the appropriate section before selecting a response.

Materials & experimental systems

n/a	Involved in the study	
☐	☒	Antibodies
☐	☒	Eukaryotic cell lines
☒	☐	Palaeontology
☐	☒	Animals and other organisms
☐	☒	Human research participants
☒	☐	Clinical data

Methods

n/a	Involved in the study	
☒	☐	ChIP-seq
☐	☒	Flow cytometry
☒	☐	MRI-based neuroimaging

Antibodies

Antibodies used

Flow cytometry antibodies were purchased from Biolegend: FITC anti-mouse CD4 (Clone# RM4-5, Cat# 100510), FITC anti-mouse B220 (Clone#RA3-6B2, Cat#103206), FITC anti-mouse F4/80 (Clone#BM8, Cat#123108), FITC anti-mouse IFN-γ (XMG1.2, #505806), FITC anti-mouse NK1.1 (Clone#PK136, Cat#108706), FITC anti-mouse CD3e (Clone#145-2C11, Cat#100306), FITC anti-mouse CD11b (Clone#M1/70, Cat# 101206), FITC anti-mouse CD11c (Clone#N418, Cat#117306), Alexa Fluor@488 anti-mouse Gr1 (Clone# RB6-8C5, Cat#108419), PE/Cy7 anti-mouse IL17A (Clone#TC11-18H10.1, Cat#506922), PE/Cy7 anti-mouse Thy1 (Clone#30-H12, Cat#105326), BV421 anti-mouse CD4(Clone#RM4-5, Cat#100544), APC anti-mouse IL-10 (Cat#JES5-16E3, Cat#505010), PE anti-mouse pStat3 (Tyr705) (Clone#13A3-1, Cat#651004), BV421 anti-mouse TCRb (Clone#H57-597, Cat#109230), APC anti-mouse IL-17 (Clone#TC11-18H10.1, Cat#506916), PE/Cy7 anti-human CD4 (Clone#A161A1, Cat#357409), PE anti-human IL-22 (Cat#2G12A41, Cat#366703); or from Invitrogen: PE anti-mouse IL-22 antibody (Clone#1H8PWSR, Cat#12-7221-82), APC anti-mouse Foxp3 (Cat#FJK-16S, Cat#17-5773-82), APC anti-mouse IL23p19 (Clone#fc23cpg, Cat#50-7023-82), Percp-eFlur710 anti-mouse pmTOR (Ser2448) (Clone#MRRBY, Cat#46-9718-42); APC anti-mouse Rorgt (Clone#B2D, Cat#17-6981-82)or R&D: APC anti-mouse HIF1α (Cat#IC1935A); or CST: Pacific blue anti-mouse pS6 Ribosomal Protein (Ser235/236) (Cat# 8520). For blocking in flow cytometry, anti-mouse CD16/32 antibody (Clone#93, Cat#101302) was purchased from Biolegend.

Westernblot antibodies were purchased from Cell Signaling Technology: Antibody against phosphorylated STAT3 (Y705, Cat#9145), phosphorylated mTOR (S2448, Cat#2971), Stat3 (D3Z2G, Cat#12640), mTOR (Cat#2972), β-actin (D6A8, Cat#8457), HIF1α (D2U3T, Cat#14179), and anti-rabbit secondary antibody conjugated with HRP (Cat#7074); or from R&D: antibody against HIF2α (Cat#AF2997), AhR (Cat#AF6697), anti-goat secondary antibody conjugated with HRP (Cat#HAF019), and anti-sheep secondary antibody conjugated with HRP (Cat#HAF016).

Chip antibodies were purchased from ActiveMotif: antibody against H3K9me3 (Cat#39161), and H3K9ac (Cat#391372), or from Cell Signaling Technology: antibody against IgG isotype control (DA1E) (Cat#3900), or from Novus Biologicals: antibody against HIF1α (Cat#NB100-134).

Antibodies for in vitro T cell culture were purchased from Bio X cell: anti-mouse IL-4 (Clone#11B1, Cat#BE0045), anti-mouse IFNg (Clone#XMG1.2, Cat#BE0055), anti-mouse CD28 (Clone#37.51, Cat# BE0015-1), and anti-mouse CD3 (Clone#145-2C11, Cat#BE0001-1); or Biolegend: anti-human CD3 (Clone#HIT3a, Cat#300302), anti-human CD28 (Clone#CD28.2, Cat#302901).

Antibodies used for depletion in vivo were purchased from Bio X cell: anti-mouse CD4 (Clone#GK1.5, Cat#BP0003-1), and anti-mouse Thy1 (Clone#30H12, Cat#BE0066)

Validation

All the antibodies were validated by manufacturer.
Flow cytometry antibodies:
FITC anti-mouse CD4: Species reactivity, mouse; Application, flow cytometry.
FITC anti-mouse B220: Species reactivity, mouse, human, cat; Application: flow cytometry.
FITC anti-mouse F4/80, Species reactivity, mouse; Application, flow cytometry.
FITC anti-mouse IFN-γ, Species reactivity, mouse; Application, flow cytometry.
FITC anti-mouse NK1.1, Species reactivity, mouse; Application, flow cytometry.
FITC anti-mouse CD3e, Species reactivity, mouse; Application, flow cytometry.
FITC anti-mouse CD11b, Species reactivity, Mouse, Human, Cross-Reactivity: Chimpanzee, Baboon, Cynomolgus, Rhesus, Rabbit (Lapine); Application, flow cytometry.
FITC anti-mouse CD11c, Species reactivity, mouse; Application, flow cytometry.
Alexa Fluor@488 anti-mouse Gr1, Species reactivity, mouse; Application, flow cytometry.
PE/Cy7 anti-mouse IL17A, Species reactivity, mouse; Application, flow cytometry.
PE/Cy7 anti-mouse Thy1, Species reactivity, mouse; Application, flow cytometry.
BV421 anti-mouse CD4, Species reactivity, mouse; Application, flow cytometry.
APC anti-mouse IL-10, Species reactivity, mouse; Application, flow cytometry.
PE anti-mouse pStat3 (Tyr705), Species reactivity, mouse, human; Application, flow cytometry.
BV421 anti-mouse TCRb, Species reactivity, mouse; Application, flow cytometry.
APC anti-mouse IL-17, Species reactivity, mouse; Application, flow cytometry.
PE/Cy7 anti-human CD4, Species reactivity, human; Application, flow cytometry.
PE anti-human IL-22, Species reactivity, human; Application, flow cytometry.
PE anti-mouse IL-22 antibody, Species reactivity, mouse; Application, flow cytometry.
APC anti-mouse Foxp3, Species reactivity, Bovine, Dog, Cat, Mouse, Pig, Rat; Application, flow cytometry.
APC anti-mouse IL23p19, Species reactivity, mouse; Application, flow cytometry.
Percp-eFlur710 anti-mouse pmTOR (Ser2448), Species reactivity, mouse and human; Application, flow cytometry.
APC anti-mouse Rorgt, Species reactivity, mouse; Application, flow cytometry, immunohistochemistry.
APC anti-mouse HIF1α, Species reactivity, mouse, human; Application, flow cytometry.
Pacific blue anti-mouse pS6 Ribosomal Protein (Ser235/236), Species reactivity, human, mice, rat, monkey, mink, S. cerevisiae; Application, flow cytometry.
Anti-mouse CD16/32 antibody, Species reactivity, mouse; Application, flow cytometry, blocking, Immunoprecipitation.
Westernblot antibodies:
Anti-phosphorylated STAT3 (Y705), Species reactivity, human, mouse, rat, monkey; Application, Western Blotting, Immunoprecipitation, IHC-Leica® Bond™, Immunohistochemistry, Immunofluorescence (Immunocytochemistry), Flow Cytometry, Chromatin IP, Chromatin IP-seq.
Anti-phosphorylated mTOR (S2448), Species reactivity, human, mouse, rat, monkey; Application, Western Blotting.
Anti-Stat3 (D372G): Species reactivity, human, mouse, rat, monkey; Application, Western Blotting, Immunoprecipitation, Immunofluorescence (Immunocytochemistry), Flow Cytometry, Chromatin IP, Chromatin IP-seq.
Anti-mTOR, Species reactivity, human, mouse, rat, monkey; Application, Western Blotting, Immunoprecipitation.
Anti-β-actin, Species reactivity, human, mouse, rat, monkey, D. melanogaster, Zebrafish; Application, Western Blotting, Immunofluorescence (Immunocytochemistry), Flow Cytometry.
Anti-HIF1α, Species reactivity, human, mouse, rat, monkey; Application, Western Blotting, Chromatin IP, Chromatin IP-seq.
Anti-Hif2α, Species reactivity, human, mouse, rat; Application, Western Blotting, Immunohistochemistry.
Anti-AhR, Species reactivity, mouse; Application, Western Blotting, Immunohistochemistry.
Chip antibodies:
Anti-H3K9me3, Species reactivity, Fission Yeast, Human, Wide Range Predicted; Application, Chromatin IP, Chromatin IP-seq, Immunofluorescence (Immunocytochemistry), Western Blotting.
Anti-H3K9ac, Species reactivity, Human, Mouse, Wide Range Predicted; Application, Chromatin IP, Chromatin IP-seq, Immunofluorescence, Western Blotting.
Anti-IgG isotype control (DA1E): Applications, Immunoprecipitation, Immunohistochemistry, Immunofluorescence, flow cytometry, Chromatin IP.
Anti-HIF1α: Species reactivity, human, mouse, rat, Canine, Guinea Pig, Primate, Xenopus, Zebrafish; Application, Western Blot, Simple Western, ELISA, Gel super shift assay, Immunoblotting, Immunofluorescence (Immunocytochemistry), Immunohistochemistry, Immunoprecipitation, Chromatin IP, Dual RNAscope ISH-IHC, knockout validated.
Antibodies for in vitro T cell culture
Anti-mouse IL-4: Species reactivity, mouse; Application, in vivo IL-4 neutralization, in vitro IL-4 neutralization, in vivo IL-4 receptor stimulation (as a complex with IL-4), Flow cytometry, Western blot.
Anti-mouse IFNg: Species reactivity, mouse; Application, in vivo IFNγ neutralization, in vitro IFNγ neutralization, ELISPOT, Flow cytometry, Western blot.
anti-mouse CD28: Species reactivity, mouse; Application, in vitro T cell stimulation/activation, in vivo CD28 blockade.
Anti-mouse CD3: Species reactivity, mouse; Application, in vitro T cell stimulation/activation, Immunofluorescence, Flow cytometry, Western blot, in vivo T cell depletion.
Anti-human CD3: Species reactivity, human; Application: Flow cytometry, Immunoprecipitation, Immunohistochemistry, activation.
Anti-human CD28: Species reactivity, Human, Baboon, Capuchin Monkey, Chimpanzee, Cynomolgus, Pigtailed Macaque, Rhesus, Sooty Mangabey, Squirrel Monkey; Application, Flow cytometry, Immunohistochemistry, Costimulation, Functional assay.
Antibodies used for depletion in vivo

Anti-mouse CD4: Species reactivity, mouse; Application: in vivo CD4+ T cell depletion, Flow cytometry, Western blot.
Anti-mouse Thy1: Species reactivity, mouse; Application, in vivo ILC depletion, in vivo T cell depletion, Western blot.

Eukaryotic cell lines

Policy information about cell lines

Cell line source(s)	Raw 264.7 (ATCC® TIB-71™) was bought from ATCC.
Authentication	Cell lines were authenticated using short tandem repeat (STR) analysis.
Mycoplasma contamination	This cell line tested negative for mycoplasma contamination.
Commonly misidentified lines (See ICLAC register)	No commonly misidentified cell lines were used in the study.

Animals and other organisms

Policy information about studies involving animals; ARRIVE guidelines recommended for reporting animal research

Laboratory animals	Wild- type C57BL/6J (B6) mice, B6.129-Prdm1tm1Clme/J (Prdm1fl/fl) mice, B6.129S1-Stat3tm1Xyfu/J (Stat3fl/fl) mice, and B6.CD4cre mice were from Jackson Laboratory. Gpr43-/- (Ffar2tm1Lex) mice were obtained from Bristol-Myers Squibb. B6 IL-22-/- mice were obtained from Amegen (Thousand Oaks, CA). CD4creHIF1αfl/fl mice were obtained from Dr. Fan Pan at Sidney Kimmel Comprehensive Cancer Center. GPR109a-/- mice were obtained from Dr. Nagendra Singh of the Augusta University. CBir1 TCR transgenic (CBir1 Tg) mice were bred n the Animal Resource Center of University of thTexas Medical Branch (UTMB). All the mice were maintained on a 12 hour-light/dark cycle and at the temperature of 20-26ȼ with 30-70% humidity in the specific pathogen free animal facilities. The experimental mice were sex-matched littermates and cohoused after weaning, and both male and females were used at 6-12 weeks.
Wild animals	This study did not involve wild animals.
Field-collected samples	This study did not involve the use of the field-collected samples.
Ethics oversight	The animal use and care were in accordance with institutional guidelines of UTMB, and all experiments were approved by the Institutional Animal Care and Use Committee of the University of Texas Medical Branch.

Note that full information on the approval of the study protocol must also be provided in the manuscript.

Human research participants

Policy information about studies involving human research participants

Population characteristics	Demographics of all the participants in this study were provided in table S1.
Recruitment	the participants were recruited with no self-selection bias or other biases.
Ethics oversight	All the human studies were approved by the Institutional Review Board for Clinical Research of Shanghai Tenth People's Hospital, Tongji University.

Note that full information on the approval of the study protocol must also be provided in the manuscript.

Flow Cytometry

Plots

Confirm that:

- [x] The axis labels state the marker and fluorochrome used (e.g. CD4-FITC).
- [x] The axis scales are clearly visible. Include numbers along axes only for bottom left plot of group (a 'group' is an analysis of identical markers).
- [x] All plots are contour plots with outliers or pseudocolor plots.
- [x] A numerical value for number of cells or percentage (with statistics) is provided.

Methodology

Sample preparation	The information was included in the Methods section.
Instrument	BD LSRFortessa (BD Biosciences)
Software	FACS DIVA software 8.0.1 (BD Biosience) and FlowJo 10.0 software (TreeStar)

Cell population abundance	There was no cell sorting in this study.
Gating strategy	The gating strategy will be presented in Extended data.

☒ Tick this box to confirm that a figure exemplifying the gating strategy is provided in the Supplementary Information.
